# (*E*)-2-[4-(Diethyl­amino)­styr­yl]-1-methyl­pyridinium 4-meth­oxy­benzene­sulfonate monohydrate

**DOI:** 10.1107/S1600536812035258

**Published:** 2012-08-15

**Authors:** Suchada Chantrapromma, Narissara Kaewmanee, Nawong Boonnak, Teerasak Anantapong, Hoong-Kun Fun

**Affiliations:** a Crystal Materials Research Unit, Department of Chemistry, Faculty of Science, Prince of Songkla University, Hat-Yai, Songkhla 90112, Thailand; b Department of Biotechnology, Faculty of Agro-Industry, Prince of Songkla University, Hat-Yai, Songkhla 90112, Thailand; c X-ray Crystallography Unit, School of Physics, Universiti Sains Malaysia, 11800 USM, Penang, Malaysia

## Abstract

In the cation of the title compound, C_18_H_23_N_2_^+^·C_7_H_7_O_4_S^−^·H_2_O, one ethyl group of the diethyl­amino unit is disordered over two sets of sites in a 0.665 (6):0.335 (6) ratio. The styrylpyridinium unit is nearly planar, with a dihedral angle between the pyridinium and benzene rings of 4.27 (8)°. In the crystal, the anion ring is almost perpendicular to the aromatic rings of the cation; the sulfonate-substituted benzene ring forms dihedral angles of 89.60 (8) and 89.37 (8)°, respectively, with the pyridinium and benzene rings of the cation. In the crystal, the three components are linked into a three-dimensional network by O—H⋯O and C—H⋯O hydrogen bonds. π–π inter­actions with centroid–centroid distances of 3.6999 (9) and 3.7106 (9) Å are also present.

## Related literature

For bond-length data, see: Allen *et al.* (1987 ▶). For background to and applications of quaternary ammonium compounds, see: Chanawanno *et al.* (2010 ▶); Domagk (1935 ▶). For related structures, see: Fun *et al.* (2011*a* ▶,*b* ▶); Kaewmanee *et al.* (2010 ▶).
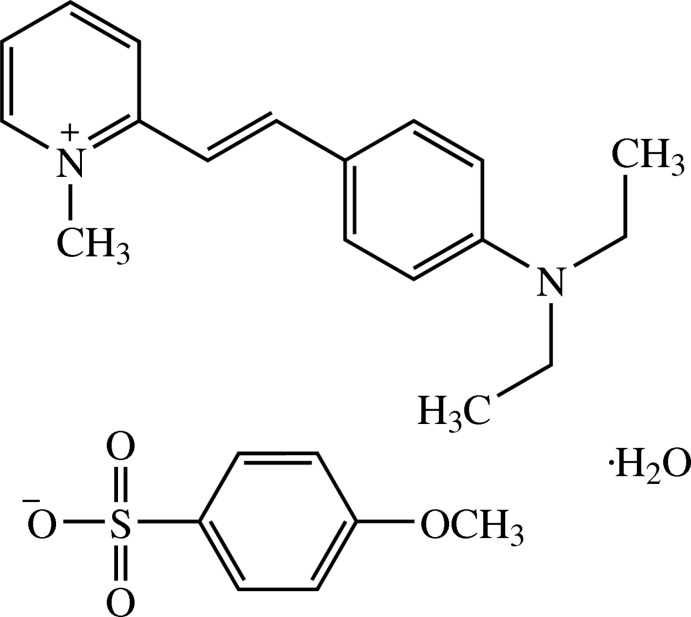


## Experimental

### 

#### Crystal data

C_18_H_23_N_2_^+^·C_7_H_7_O_4_S^−^·H_2_O
*M* *_r_* = 472.60
Triclinic, 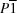

*a* = 7.4430 (2) Å
*b* = 10.3298 (2) Å
*c* = 16.3817 (4) Å
α = 91.265 (1)°
β = 100.794 (1)°
γ = 102.281 (1)°
*V* = 1206.39 (5) Å^3^
*Z* = 2
Mo *K*α radiation
μ = 0.17 mm^−1^
*T* = 298 K
0.53 × 0.19 × 0.13 mm

#### Data collection

Bruker APEXII CCD area-detector diffractometer
Absorption correction: multi-scan (*SADABS*; Bruker, 2009 ▶) *T*_min_ = 0.914, *T*_max_ = 0.978
28598 measured reflections
6988 independent reflections
4465 reflections with *I* > 2σ(*I*)
*R*_int_ = 0.029

#### Refinement

*R*[*F* ^2^ > 2σ(*F* ^2^)] = 0.049
*wR*(*F* ^2^) = 0.145
*S* = 1.06
6988 reflections
312 parameters
H-atom parameters constrained
Δρ_max_ = 0.29 e Å^−3^
Δρ_min_ = −0.32 e Å^−3^

### 

Data collection: *APEX2* (Bruker, 2009 ▶); cell refinement: *SAINT* (Bruker, 2009 ▶); data reduction: *SAINT*; program(s) used to solve structure: *SHELXTL* (Sheldrick, 2008 ▶); program(s) used to refine structure: *SHELXTL*; molecular graphics: *SHELXTL*; software used to prepare material for publication: *SHELXTL* and *PLATON* (Spek, 2009 ▶).

## Supplementary Material

Crystal structure: contains datablock(s) global, I. DOI: 10.1107/S1600536812035258/is5179sup1.cif

Structure factors: contains datablock(s) I. DOI: 10.1107/S1600536812035258/is5179Isup2.hkl

Supplementary material file. DOI: 10.1107/S1600536812035258/is5179Isup3.cml

Additional supplementary materials:  crystallographic information; 3D view; checkCIF report

## Figures and Tables

**Table 1:** Hydrogen-bond geometry (Å, °)

*D*—H⋯*A*	*D*—H	H⋯*A*	*D*⋯*A*	*D*—H⋯*A*
O1*W*—H1*W*1⋯O2^i^	0.99	2.53	3.371 (2)	143
O1*W*—H1*W*1⋯O3^i^	0.99	2.15	3.073 (2)	155
O1*W*—H2*W*1⋯O2^ii^	0.89	1.90	2.791 (2)	176
C1—H1*A*⋯O3^iii^	0.93	2.54	3.419 (2)	158
C2—H2*A*⋯O3^iv^	0.93	2.47	3.349 (2)	158
C4—H4*A*⋯O1*W* ^v^	0.93	2.47	3.381 (2)	166
C7—H7*A*⋯O1*W* ^v^	0.93	2.58	3.479 (2)	163
C17—H17*A*⋯O1^i^	0.96	2.58	3.435 (3)	149
C18—H18*A*⋯O3^iii^	0.96	2.54	3.466 (2)	162
C18—H18*B*⋯O4^vi^	0.96	2.47	3.221 (2)	135

Symmetry codes: (i) 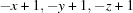; (ii) 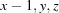; (iii) 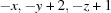; (iv) 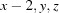; (v) 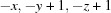; (vi) 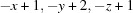.

**Fig. 1.**
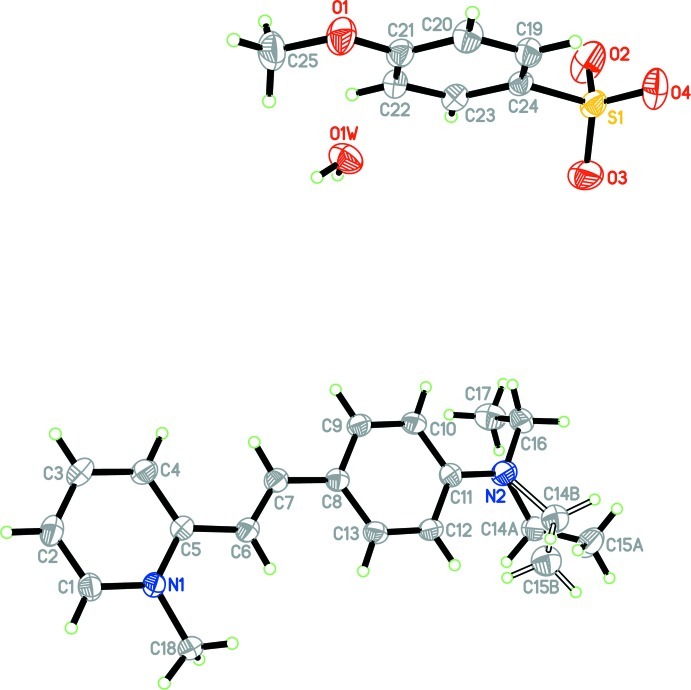
The asymmetric unit of the title compound, showing 30% probability displacement ellipsoids and the atom-numbering scheme. Open bonds show the minor component.

**Fig. 2.**
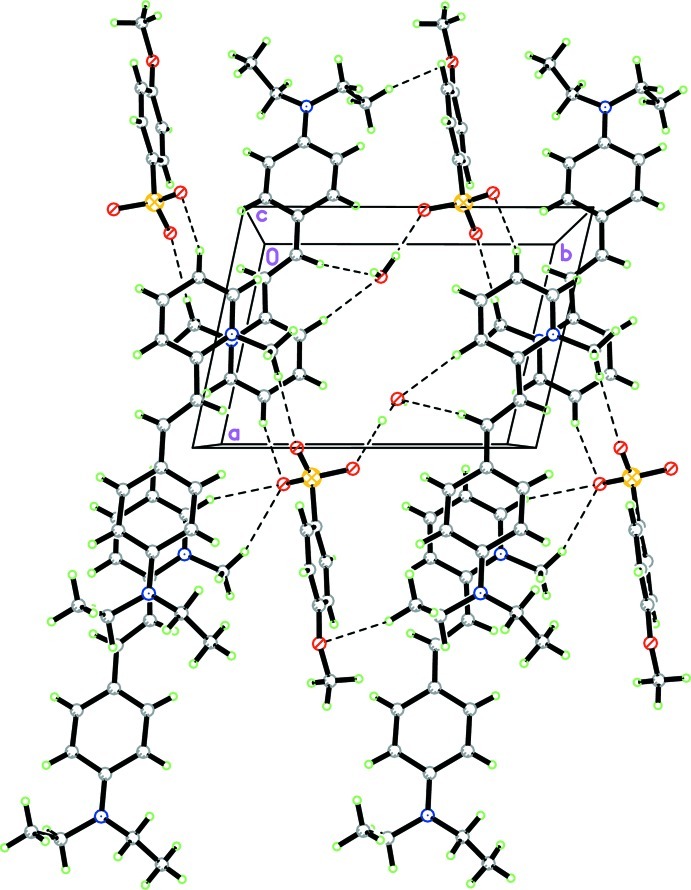
A crystal packing diagram of the major component viewed along the c axis. The O—H···O hydrogen bonds and weak C—H···O interactions are drawn as dashed lines.

## supplementary crystallographic information

### Comment

For a long time, quaternary ammonium compounds (QACs) have been used as disinfectants in both medical and domestic purposes due to their low toxicity and wide-ranging antimicrobial properties (Domagk, 1935). We have during the course of our research reported on the synthesis and antibacterial activity of pyridinium derivatives (Chanawanno *et al.*, 2010). The title compound (I) was synthesized and tested for antibacterial activity. Our antibacterial assay showed that (I) exhibits moderate activity against *Pseudomonas aeruginosa* with the MIC value of 37.5 µg/ml. Herein its crystal structure is reported.

The asymmetric unit of the title compound (Fig. 1) consists of a C_18_H_23_N_2_^+^ cation, a C_7_H_7_O_4_S^-^ anion and one H_2_O molecule. The cation exists in the *E* configuration with respect to the C6═C7 double bond [1.332 (2) Å] and the torsion angle C5–C6–C7–C8 = -177.25 (15)°. The cation is nearly planar with the dihedral angle between the C1–C5/N1 pyridinium and the C8–C13 benzene rings being 4.27 (8)°. One ethyl unit of the diethylamino moiety is disordered over two positions; the major component *A* and the minor component *B* (Fig. 1), with the refined site-occupancy ratio of 0.665 (6)/0.335 (6). The diethylamino moiety with the disordered ethyl unit deviated from the attached benzene ring and the disordered ethyl units point opposite to each other as indicated by the torsion angles C11–N2–C14A–C15A = -103.6 (3)° for the major component *A* and C11–N2–C14B–C15B = 101.7 (5)° for the minor component *B*. The other diethylamino moiety also deviated from its bound benzene ring with the torsion angle C11–N2–C16–C17 = -80.5 (2)° and point toward the same direction as the ethyl unit of the minor component *B*. The cation and anion are inclined to each other as indicated by the dihedral angles between the pyridinium and benzene rings of cation, and the sulfonate substituted benzene ring being 89.60 (8) and 89.37 (8)°, respectively. The bond lengths are in normal ranges (Allen *et al.*, 1987) and comparable with related structures (Fun *et al.*, 2011*a*,*b*)

In the crystal packing, the cations, anions and water molecules are linked into a network by O—H···O hydrogen bonds and C—H···O weak interactions (Fig. 2 and Table 1). π···π interactions with the centroid–centroid distances of *Cg*1···*Cg*1^vii^ = 3.7106 (9) Å and *Cg*1···*Cg*2^ii^ = 3.6999 (9) Å were observed; *Cg*1 and *Cg*2 are the centroids of N1/C1–C5 and C8–C13 rings, respectively.

### Experimental

A solution of 2-[(*E*)-4-(diethylamino)styryl]-1-methylpyridinium iodide (0.13 g, 0.33 mmol) (Kaewmanee *et al.*, 2010) in hot methanol (20 ml) was mixed with a solution of silver(I) 4-methoxybenzenesulfonate (0.10 g, 0.33 mmol) in hot methanol (20 ml). The mixture immediately yielded a grey precipitate of silver iodide. After stirring the mixture for 30 min, the precipitate of silver iodide was removed and the resulting solution was evaporated yielding a deep orange solid. Orange single crystals of the title compound suitable for *X*-ray structure determination were recrystallized from methanol by slow evaporation of the solvent at room temperature after a few weeks (m.p. 421–423 K).

### Refinement

All H atoms were positioned geometrically and allowed to ride on their parent atoms, with O—H = 0.89 and 0.99 Å, C—H = 0.93 Å for aromatic and CH and 0.96 Å for CH_3_ atoms. The *U*_iso_(H) values were constrained to be 1.5*U*_eq_ of the carrier atom for methyl H atoms and 1.2*U*_eq_ for the remaining H atoms. A rotating group model was used for the methyl groups. One ethyl unit of the diethylamino is disordered over two sites with refined site occupancies of 0.665 (6) and 0.335 (6).

### Crystal data

**Table:**

C_18_H_23_N_2_^+^·C_7_H_7_O_4_S^−^·H_2_O	*Z* = 2
*M**_r_* = 472.60	*F*(000) = 504
Triclinic, *P*1	*D*_x_ = 1.301 Mg m^−^^3^
Hall symbol: -P 1	Melting point = 421–423 K
*a* = 7.4430 (2) Å	Mo *K*α radiation, λ = 0.71073 Å
*b* = 10.3298 (2) Å	Cell parameters from 6988 reflections
*c* = 16.3817 (4) Å	θ = 2.0–30.0°
α = 91.265 (1)°	µ = 0.17 mm^−^^1^
β = 100.794 (1)°	*T* = 298 K
γ = 102.281 (1)°	Needle, orange
*V* = 1206.39 (5) Å^3^	0.53 × 0.19 × 0.13 mm

### Data collection

**Table:**

Bruker APEXII CCD area-detector diffractometer	6988 independent reflections
Radiation source: sealed tube	4465 reflections with *I* > 2σ(*I*)
Graphite monochromator	*R*_int_ = 0.029
φ and ω scans	θ_max_ = 30.0°, θ_min_ = 2.0°
Absorption correction: multi-scan (*SADABS*; Bruker, 2009)	*h* = −10→10
*T*_min_ = 0.914, *T*_max_ = 0.978	*k* = −14→14
28598 measured reflections	*l* = −23→23

### Refinement

**Table:**

Refinement on *F*^2^	Primary atom site location: structure-invariant direct methods
Least-squares matrix: full	Secondary atom site location: difference Fourier map
*R*[*F*^2^ > 2σ(*F*^2^)] = 0.049	Hydrogen site location: inferred from neighbouring sites
*wR*(*F*^2^) = 0.145	H-atom parameters constrained
*S* = 1.06	*w* = 1/[σ^2^(*F*_o_^2^) + (0.0616*P*)^2^ + 0.1741*P*] where *P* = (*F*_o_^2^ + 2*F*_c_^2^)/3
6988 reflections	(Δ/σ)_max_ = 0.001
312 parameters	Δρ_max_ = 0.29 e Å^−^^3^
0 restraints	Δρ_min_ = −0.32 e Å^−^^3^

### Special details

**Table:**

Geometry. All e.s.d.'s (except the e.s.d. in the dihedral angle between two l.s. planes) are estimated using the full covariance matrix. The cell e.s.d.'s are taken into account individually in the estimation of e.s.d.'s in distances, angles and torsion angles; correlations between e.s.d.'s in cell parameters are only used when they are defined by crystal symmetry. An approximate (isotropic) treatment of cell e.s.d.'s is used for estimating e.s.d.'s involving l.s. planes.
Refinement. Refinement of *F*^2^ against ALL reflections. The weighted *R*-factor *wR* and goodness of fit *S* are based on *F*^2^, conventional *R*-factors *R* are based on *F*, with *F* set to zero for negative *F*^2^. The threshold expression of *F*^2^ > σ(*F*^2^) is used only for calculating *R*-factors(gt) *etc*. and is not relevant to the choice of reflections for refinement. *R*-factors based on *F*^2^ are statistically about twice as large as those based on *F*, and *R*- factors based on ALL data will be even larger.

### Fractional atomic coordinates and isotropic or equivalent isotropic displacement parameters (Å^2^)

**Table:**

	*x*	*y*	*z*	*U*_iso_*/*U*_eq_	Occ. (<1)
N1	−0.50562 (17)	1.00872 (12)	0.61471 (8)	0.0443 (3)	
N2	0.6382 (2)	0.95002 (15)	0.87724 (11)	0.0724 (5)	
C1	−0.6858 (2)	0.98155 (17)	0.57237 (10)	0.0522 (4)	
H1A	−0.7540	1.0476	0.5693	0.063*	
C2	−0.7688 (2)	0.85902 (18)	0.53425 (11)	0.0594 (4)	
H2A	−0.8923	0.8413	0.5053	0.071*	
C3	−0.6657 (3)	0.76154 (18)	0.53944 (11)	0.0615 (5)	
H3A	−0.7199	0.6772	0.5142	0.074*	
C4	−0.4839 (2)	0.78983 (16)	0.58190 (11)	0.0548 (4)	
H4A	−0.4149	0.7243	0.5845	0.066*	
C5	−0.3994 (2)	0.91516 (15)	0.62153 (9)	0.0448 (3)	
C6	−0.2092 (2)	0.94942 (16)	0.66919 (10)	0.0499 (4)	
H6A	−0.1625	1.0363	0.6914	0.060*	
C7	−0.0969 (2)	0.86422 (16)	0.68320 (10)	0.0494 (4)	
H7A	−0.1447	0.7789	0.6583	0.059*	
C8	0.0914 (2)	0.88967 (15)	0.73292 (9)	0.0456 (3)	
C9	0.1915 (2)	0.78913 (16)	0.74038 (10)	0.0508 (4)	
H9A	0.1356	0.7070	0.7124	0.061*	
C10	0.3691 (2)	0.80681 (16)	0.78742 (11)	0.0524 (4)	
H10A	0.4304	0.7369	0.7907	0.063*	
C11	0.4595 (2)	0.92947 (16)	0.83076 (10)	0.0509 (4)	
C12	0.3604 (2)	1.03119 (16)	0.82244 (10)	0.0519 (4)	
H12A	0.4165	1.1138	0.8498	0.062*	
C13	0.1826 (2)	1.01232 (16)	0.77504 (10)	0.0495 (4)	
H13A	0.1217	1.0824	0.7709	0.059*	
C16	0.7364 (3)	0.84251 (19)	0.89166 (13)	0.0641 (5)	
H16A	0.8703	0.8796	0.9060	0.077*	
H16B	0.7119	0.7867	0.8407	0.077*	
C17	0.6778 (3)	0.7585 (2)	0.96027 (14)	0.0828 (6)	
H17A	0.7471	0.6898	0.9679	0.124*	
H17B	0.5461	0.7192	0.9456	0.124*	
H17C	0.7029	0.8131	1.0111	0.124*	
C18	−0.4297 (2)	1.14455 (15)	0.65430 (11)	0.0539 (4)	
H18A	−0.5196	1.1983	0.6384	0.081*	
H18B	−0.3154	1.1828	0.6364	0.081*	
H18C	−0.4050	1.1406	0.7137	0.081*	
S1	0.85883 (5)	0.64249 (4)	0.33983 (3)	0.05481 (14)	
O1	0.14269 (17)	0.50219 (14)	0.09686 (8)	0.0711 (4)	
O2	0.8984 (2)	0.52093 (14)	0.37195 (11)	0.0983 (6)	
O3	0.8267 (2)	0.72821 (16)	0.40326 (10)	0.0928 (5)	
O4	0.99412 (18)	0.70754 (16)	0.29340 (10)	0.0853 (4)	
C19	0.6343 (2)	0.61781 (16)	0.18441 (11)	0.0535 (4)	
H19A	0.7427	0.6557	0.1655	0.064*	
C20	0.4652 (2)	0.58362 (18)	0.12927 (11)	0.0591 (4)	
H20A	0.4600	0.5972	0.0730	0.071*	
C21	0.3033 (2)	0.52931 (16)	0.15715 (10)	0.0518 (4)	
C22	0.3100 (2)	0.50668 (16)	0.24013 (11)	0.0528 (4)	
H22A	0.2010	0.4699	0.2590	0.063*	
C23	0.4813 (2)	0.53940 (15)	0.29535 (10)	0.0504 (4)	
H23A	0.4871	0.5232	0.3513	0.061*	
C24	0.6431 (2)	0.59581 (14)	0.26792 (10)	0.0446 (3)	
C25	−0.0281 (3)	0.4442 (3)	0.12097 (15)	0.0874 (7)	
H25A	−0.1299	0.4370	0.0742	0.131*	
H25B	−0.0474	0.4989	0.1653	0.131*	
H25C	−0.0228	0.3575	0.1397	0.131*	
C14A	0.7158 (4)	1.0661 (3)	0.9384 (2)	0.0620 (10)	0.665 (7)
H14A	0.7805	1.0379	0.9897	0.074*	0.665 (7)
H14B	0.6145	1.1036	0.9511	0.074*	0.665 (7)
C15A	0.8500 (5)	1.1693 (4)	0.9030 (2)	0.0837 (13)	0.665 (7)
H15A	0.9023	1.2430	0.9433	0.126*	0.665 (7)
H15B	0.7844	1.1994	0.8534	0.126*	0.665 (7)
H15C	0.9489	1.1314	0.8897	0.126*	0.665 (7)
C14B	0.7614 (10)	1.0907 (7)	0.8914 (5)	0.067 (2)*	0.335 (7)
H14C	0.8900	1.0911	0.8876	0.080*	0.335 (7)
H14D	0.7132	1.1497	0.8520	0.080*	0.335 (7)
C15B	0.7461 (11)	1.1271 (9)	0.9771 (5)	0.085 (3)*	0.335 (7)
H15D	0.8186	1.2155	0.9935	0.127*	0.335 (7)
H15E	0.7928	1.0661	1.0144	0.127*	0.335 (7)
H15F	0.6171	1.1235	0.9792	0.127*	0.335 (7)
O1W	0.2163 (2)	0.42954 (15)	0.44206 (9)	0.0802 (4)	
H1W1	0.2043	0.4060	0.4991	0.096*	
H2W1	0.1179	0.4626	0.4195	0.096*	

### Atomic displacement parameters (Å^2^)

**Table:**

	*U*^11^	*U*^22^	*U*^33^	*U*^12^	*U*^13^	*U*^23^
N1	0.0394 (6)	0.0452 (7)	0.0455 (7)	0.0056 (5)	0.0063 (5)	−0.0013 (5)
N2	0.0520 (8)	0.0551 (9)	0.1020 (13)	0.0181 (7)	−0.0109 (8)	−0.0106 (8)
C1	0.0411 (8)	0.0600 (10)	0.0536 (9)	0.0101 (7)	0.0061 (7)	0.0001 (7)
C2	0.0443 (8)	0.0672 (11)	0.0569 (10)	−0.0012 (8)	0.0018 (7)	−0.0034 (8)
C3	0.0620 (10)	0.0512 (9)	0.0599 (10)	−0.0032 (8)	0.0026 (8)	−0.0072 (8)
C4	0.0582 (10)	0.0439 (8)	0.0582 (10)	0.0074 (7)	0.0058 (8)	−0.0013 (7)
C5	0.0455 (8)	0.0422 (8)	0.0448 (8)	0.0067 (6)	0.0077 (6)	0.0019 (6)
C6	0.0454 (8)	0.0453 (8)	0.0545 (9)	0.0070 (6)	0.0030 (7)	−0.0030 (7)
C7	0.0493 (8)	0.0469 (8)	0.0503 (9)	0.0100 (7)	0.0065 (7)	0.0004 (7)
C8	0.0467 (8)	0.0449 (8)	0.0455 (8)	0.0107 (6)	0.0094 (6)	0.0032 (6)
C9	0.0533 (9)	0.0422 (8)	0.0555 (9)	0.0112 (7)	0.0067 (7)	−0.0010 (7)
C10	0.0536 (9)	0.0443 (8)	0.0622 (10)	0.0186 (7)	0.0093 (8)	0.0029 (7)
C11	0.0447 (8)	0.0494 (9)	0.0579 (9)	0.0128 (7)	0.0063 (7)	0.0016 (7)
C12	0.0520 (9)	0.0432 (8)	0.0587 (10)	0.0127 (7)	0.0049 (7)	−0.0048 (7)
C13	0.0509 (9)	0.0452 (8)	0.0545 (9)	0.0169 (7)	0.0086 (7)	0.0007 (7)
C16	0.0504 (9)	0.0674 (11)	0.0759 (12)	0.0243 (8)	0.0039 (9)	−0.0013 (9)
C17	0.0948 (16)	0.0816 (14)	0.0772 (14)	0.0340 (12)	0.0137 (12)	0.0032 (11)
C18	0.0488 (9)	0.0458 (8)	0.0636 (10)	0.0095 (7)	0.0048 (7)	−0.0107 (7)
S1	0.0428 (2)	0.0440 (2)	0.0716 (3)	0.01129 (16)	−0.00529 (19)	−0.00146 (19)
O1	0.0525 (7)	0.0869 (9)	0.0627 (8)	0.0079 (6)	−0.0074 (6)	0.0003 (7)
O2	0.0759 (10)	0.0629 (9)	0.1366 (14)	0.0167 (7)	−0.0315 (9)	0.0240 (9)
O3	0.0668 (9)	0.1074 (12)	0.0924 (11)	0.0314 (8)	−0.0219 (8)	−0.0470 (9)
O4	0.0450 (7)	0.0923 (10)	0.1062 (11)	−0.0044 (7)	0.0055 (7)	0.0094 (9)
C19	0.0482 (8)	0.0485 (9)	0.0628 (10)	0.0058 (7)	0.0137 (7)	0.0038 (7)
C20	0.0594 (10)	0.0637 (11)	0.0501 (9)	0.0069 (8)	0.0076 (8)	0.0069 (8)
C21	0.0463 (8)	0.0493 (9)	0.0555 (10)	0.0103 (7)	0.0003 (7)	−0.0018 (7)
C22	0.0433 (8)	0.0525 (9)	0.0596 (10)	0.0041 (7)	0.0105 (7)	−0.0003 (7)
C23	0.0511 (9)	0.0469 (8)	0.0501 (9)	0.0073 (7)	0.0062 (7)	0.0016 (7)
C24	0.0421 (7)	0.0345 (7)	0.0547 (9)	0.0092 (6)	0.0030 (6)	−0.0014 (6)
C25	0.0442 (10)	0.1211 (19)	0.0871 (15)	0.0093 (11)	0.0008 (10)	−0.0126 (14)
C14A	0.0534 (16)	0.0638 (18)	0.064 (2)	0.0186 (13)	−0.0045 (14)	−0.0106 (15)
C15A	0.079 (2)	0.060 (2)	0.096 (3)	−0.0026 (17)	0.0013 (18)	−0.0063 (17)
O1W	0.0792 (9)	0.0933 (10)	0.0774 (9)	0.0416 (8)	0.0134 (7)	0.0030 (8)

### Geometric parameters (Å, º)

**Table:**

N1—C1	1.3572 (19)	C18—H18A	0.9600
N1—C5	1.367 (2)	C18—H18B	0.9600
N1—C18	1.4811 (19)	C18—H18C	0.9600
N2—C11	1.374 (2)	S1—O3	1.4364 (15)
N2—C16	1.454 (2)	S1—O4	1.4372 (15)
N2—C14A	1.487 (4)	S1—O2	1.4383 (14)
N2—C14B	1.531 (8)	S1—C24	1.7706 (15)
C1—C2	1.364 (2)	O1—C21	1.3732 (19)
C1—H1A	0.9300	O1—C25	1.416 (2)
C2—C3	1.385 (3)	C19—C20	1.377 (2)
C2—H2A	0.9300	C19—C24	1.383 (2)
C3—C4	1.368 (2)	C19—H19A	0.9300
C3—H3A	0.9300	C20—C21	1.380 (2)
C4—C5	1.399 (2)	C20—H20A	0.9300
C4—H4A	0.9300	C21—C22	1.378 (2)
C5—C6	1.449 (2)	C22—C23	1.389 (2)
C6—C7	1.332 (2)	C22—H22A	0.9300
C6—H6A	0.9300	C23—C24	1.381 (2)
C7—C8	1.450 (2)	C23—H23A	0.9300
C7—H7A	0.9300	C25—H25A	0.9600
C8—C9	1.396 (2)	C25—H25B	0.9600
C8—C13	1.401 (2)	C25—H25C	0.9600
C9—C10	1.373 (2)	C14A—C15A	1.504 (6)
C9—H9A	0.9300	C14A—H14A	0.9700
C10—C11	1.407 (2)	C14A—H14B	0.9700
C10—H10A	0.9300	C15A—H15A	0.9600
C11—C12	1.402 (2)	C15A—H15B	0.9600
C12—C13	1.375 (2)	C15A—H15C	0.9600
C12—H12A	0.9300	C14B—C15B	1.477 (12)
C13—H13A	0.9300	C14B—H14C	0.9700
C16—C17	1.507 (3)	C14B—H14D	0.9700
C16—H16A	0.9700	C15B—H15D	0.9600
C16—H16B	0.9700	C15B—H15E	0.9600
C17—H17A	0.9600	C15B—H15F	0.9600
C17—H17B	0.9600	O1W—H1W1	0.9855
C17—H17C	0.9600	O1W—H2W1	0.8948
			
C1—N1—C5	121.85 (13)	H17B—C17—H17C	109.5
C1—N1—C18	116.77 (13)	N1—C18—H18A	109.5
C5—N1—C18	121.38 (12)	N1—C18—H18B	109.5
C11—N2—C16	121.76 (14)	H18A—C18—H18B	109.5
C11—N2—C14A	121.37 (16)	N1—C18—H18C	109.5
C16—N2—C14A	113.64 (17)	H18A—C18—H18C	109.5
C11—N2—C14B	119.6 (3)	H18B—C18—H18C	109.5
C16—N2—C14B	115.9 (3)	O3—S1—O4	113.46 (10)
N1—C1—C2	121.17 (16)	O3—S1—O2	111.88 (11)
N1—C1—H1A	119.4	O4—S1—O2	112.97 (10)
C2—C1—H1A	119.4	O3—S1—C24	105.87 (8)
C1—C2—C3	118.79 (16)	O4—S1—C24	106.18 (8)
C1—C2—H2A	120.6	O2—S1—C24	105.71 (8)
C3—C2—H2A	120.6	C21—O1—C25	117.97 (15)
C4—C3—C2	119.71 (15)	C20—C19—C24	120.06 (15)
C4—C3—H3A	120.1	C20—C19—H19A	120.0
C2—C3—H3A	120.1	C24—C19—H19A	120.0
C3—C4—C5	121.47 (16)	C19—C20—C21	120.26 (16)
C3—C4—H4A	119.3	C19—C20—H20A	119.9
C5—C4—H4A	119.3	C21—C20—H20A	119.9
N1—C5—C4	117.00 (14)	O1—C21—C22	124.66 (15)
N1—C5—C6	119.21 (13)	O1—C21—C20	115.03 (15)
C4—C5—C6	123.79 (15)	C22—C21—C20	120.31 (15)
C7—C6—C5	124.12 (14)	C21—C22—C23	119.25 (15)
C7—C6—H6A	117.9	C21—C22—H22A	120.4
C5—C6—H6A	117.9	C23—C22—H22A	120.4
C6—C7—C8	127.23 (15)	C24—C23—C22	120.60 (15)
C6—C7—H7A	116.4	C24—C23—H23A	119.7
C8—C7—H7A	116.4	C22—C23—H23A	119.7
C9—C8—C13	116.61 (14)	C23—C24—C19	119.50 (14)
C9—C8—C7	119.95 (14)	C23—C24—S1	119.97 (12)
C13—C8—C7	123.44 (14)	C19—C24—S1	120.51 (12)
C10—C9—C8	122.58 (14)	O1—C25—H25A	109.5
C10—C9—H9A	118.7	O1—C25—H25B	109.5
C8—C9—H9A	118.7	H25A—C25—H25B	109.5
C9—C10—C11	120.76 (15)	O1—C25—H25C	109.5
C9—C10—H10A	119.6	H25A—C25—H25C	109.5
C11—C10—H10A	119.6	H25B—C25—H25C	109.5
N2—C11—C12	121.41 (14)	N2—C14A—C15A	109.7 (3)
N2—C11—C10	121.78 (15)	N2—C14A—H14A	109.7
C12—C11—C10	116.78 (14)	C15A—C14A—H14A	109.7
C13—C12—C11	121.91 (14)	N2—C14A—H14B	109.7
C13—C12—H12A	119.0	C15A—C14A—H14B	109.7
C11—C12—H12A	119.0	H14A—C14A—H14B	108.2
C12—C13—C8	121.35 (14)	C15B—C14B—N2	101.4 (6)
C12—C13—H13A	119.3	C15B—C14B—H14C	111.5
C8—C13—H13A	119.3	N2—C14B—H14C	111.5
N2—C16—C17	112.53 (17)	C15B—C14B—H14D	111.5
N2—C16—H16A	109.1	N2—C14B—H14D	111.5
C17—C16—H16A	109.1	H14C—C14B—H14D	109.3
N2—C16—H16B	109.1	C14B—C15B—H15D	109.5
C17—C16—H16B	109.1	C14B—C15B—H15E	109.5
H16A—C16—H16B	107.8	H15D—C15B—H15E	109.5
C16—C17—H17A	109.5	C14B—C15B—H15F	109.5
C16—C17—H17B	109.5	H15D—C15B—H15F	109.5
H17A—C17—H17B	109.5	H15E—C15B—H15F	109.5
C16—C17—H17C	109.5	H1W1—O1W—H2W1	108.6
H17A—C17—H17C	109.5		
			
C5—N1—C1—C2	−0.3 (2)	C9—C8—C13—C12	−1.2 (2)
C18—N1—C1—C2	−179.54 (15)	C7—C8—C13—C12	179.05 (15)
N1—C1—C2—C3	0.1 (3)	C11—N2—C16—C17	−80.5 (2)
C1—C2—C3—C4	−0.4 (3)	C14A—N2—C16—C17	79.5 (3)
C2—C3—C4—C5	0.9 (3)	C14B—N2—C16—C17	118.5 (4)
C1—N1—C5—C4	0.8 (2)	C24—C19—C20—C21	1.1 (3)
C18—N1—C5—C4	179.99 (14)	C25—O1—C21—C22	−1.4 (3)
C1—N1—C5—C6	−178.59 (14)	C25—O1—C21—C20	178.95 (18)
C18—N1—C5—C6	0.6 (2)	C19—C20—C21—O1	178.51 (15)
C3—C4—C5—N1	−1.1 (2)	C19—C20—C21—C22	−1.1 (3)
C3—C4—C5—C6	178.25 (16)	O1—C21—C22—C23	−179.48 (15)
N1—C5—C6—C7	176.57 (15)	C20—C21—C22—C23	0.1 (3)
C4—C5—C6—C7	−2.8 (3)	C21—C22—C23—C24	0.9 (2)
C5—C6—C7—C8	−177.25 (15)	C22—C23—C24—C19	−1.0 (2)
C6—C7—C8—C9	−179.16 (16)	C22—C23—C24—S1	177.67 (12)
C6—C7—C8—C13	0.6 (3)	C20—C19—C24—C23	0.0 (2)
C13—C8—C9—C10	1.1 (2)	C20—C19—C24—S1	−178.67 (13)
C7—C8—C9—C10	−179.14 (15)	O3—S1—C24—C23	−54.23 (15)
C8—C9—C10—C11	−0.1 (3)	O4—S1—C24—C23	−175.13 (13)
C16—N2—C11—C12	175.03 (17)	O2—S1—C24—C23	64.62 (15)
C14A—N2—C11—C12	16.6 (3)	O3—S1—C24—C19	124.45 (15)
C14B—N2—C11—C12	−24.6 (4)	O4—S1—C24—C19	3.56 (16)
C16—N2—C11—C10	−6.8 (3)	O2—S1—C24—C19	−116.70 (15)
C14A—N2—C11—C10	−165.2 (2)	C11—N2—C14A—C15A	−103.6 (3)
C14B—N2—C11—C10	153.6 (4)	C16—N2—C14A—C15A	96.4 (3)
C9—C10—C11—N2	−179.06 (17)	C14B—N2—C14A—C15A	−5.6 (5)
C9—C10—C11—C12	−0.8 (3)	C11—N2—C14B—C15B	101.7 (5)
N2—C11—C12—C13	178.98 (17)	C16—N2—C14B—C15B	−96.8 (5)
C10—C11—C12—C13	0.7 (3)	C14A—N2—C14B—C15B	−1.8 (4)
C11—C12—C13—C8	0.3 (3)		

### Hydrogen-bond geometry (Å, º)

**Table:**

*D*—H···*A*	*D*—H	H···*A*	*D*···*A*	*D*—H···*A*
O1*W*—H1*W*1···O2^i^	0.99	2.53	3.371 (2)	143
O1*W*—H1*W*1···O3^i^	0.99	2.15	3.073 (2)	155
O1*W*—H2*W*1···O2^ii^	0.89	1.90	2.791 (2)	176
C1—H1*A*···O3^iii^	0.93	2.54	3.419 (2)	158
C2—H2*A*···O3^iv^	0.93	2.47	3.349 (2)	158
C4—H4*A*···O1*W*^v^	0.93	2.47	3.381 (2)	166
C7—H7*A*···O1*W*^v^	0.93	2.58	3.479 (2)	163
C17—H17*A*···O1^i^	0.96	2.58	3.435 (3)	149
C18—H18*A*···O3^iii^	0.96	2.54	3.466 (2)	162
C18—H18*B*···O4^vi^	0.96	2.47	3.221 (2)	135

Symmetry codes: (i) −*x*+1, −*y*+1, −*z*+1; (ii) *x*−1, *y*, *z*; (iii) −*x*, −*y*+2, −*z*+1; (iv) *x*−2, *y*, *z*; (v) −*x*, −*y*+1, −*z*+1; (vi) −*x*+1, −*y*+2, −*z*+1.
